# An adaptive reclosing scheme for flexible DC grid based on residual voltage S-transformation

**DOI:** 10.1038/s41598-025-96571-w

**Published:** 2025-04-12

**Authors:** Yue Dai, Hongchun Shu, Na An, Yutao Tang, Yiming Han

**Affiliations:** 1 State Key Laboratory of Collaborative Innovation Center for Smart Grid Fault Detection, Protection and Control Jointly, Kunming University of Science and Technology, Kunming, 650500 China; 2 Faculty of Mechanical and Electrical Engineering, Kunming University of Science and Technology, Kunming, 650500 China

**Keywords:** Flexible DC grid, DCCB, Identification of fault nature, Adaptive reclosing, S-transform, Electrical and electronic engineering, Energy science and technology

## Abstract

When a fault occurs on a line, it is necessary to quickly isolate the fault to improve the safety and reliability of the transmission system. Due to the lack of effective ability to identify the nature of faults, the traditional automatic reclosing scheme is highly likely to cause the circuit breaker to reclose onto a permanent fault in practical applications. This will then cause a secondary impact on the power transmission system, seriously threatening the stable operation of the system. Furthermore, existing methods lack sufficient accuracy in determining fault arc extinction moments, making it difficult to meet the urgent need for rapid power supply restoration. In view of the above problems, this paper innovatively proposes an adaptive reclosing scheme based on the S-transform of the residual voltage. This scheme cleverly applies the principle of electrostatic induction to accurately calculate the induced voltage generated by the healthy pole line on the faulty pole line before and after the fault arc extinction, and then discovers the key characteristic that after the fault arc extinction, the voltage of the faulty pole will deviate from the zero axis due to the effect of the induced voltage. Based on this important discovery, this paper conducts an S-transform processing on the residual voltage of the faulty pole, and further enhances the abrupt change characteristics of the residual voltage by calculating the sum of the amplitude squares and the mean value, thus achieving the accurate identification of the fault nature and the precise determination of the arc extinction moment. It is particularly worth mentioning that this method does not require the addition of extra control measures during the implementation process, and has high convenience and practicality. Through simulation experiments for verification, the results clearly show that compared with the traditional automatic reclosing scheme, when facing transient faults, the scheme proposed in this paper can shorten the reclosing time, and thus effectively accelerate the speed of the system’s power supply restoration. At the same time, this scheme can also effectively prevent the secondary fault impact on the system caused by the DC circuit breaker reclosing onto a permanent fault, providing a more reliable guarantee for the safe and stable operation of the power transmission system.

## Introduction

In recent years, new energy in the central and western regions of China has developed rapidly. The high proportion of renewable energy determines the need for more flexible grid connection technology in the new power system^1–3^. From the perspective of operational flexibility of transmission system and reliability of power supply, flexible DC network with multiple power supplies and multiple drop points of receiving power has become one of the development trends of new power system^4,5^, but it also has the disadvantages of complex structure and high failure probability. To ensure safe and reliable operation of the power grid, after a fault occurs on the DC line, the protection device needs to quickly isolate the fault and restore power supply as soon as possible.

In the flexible DC power grid, the line protection and recovery schemes are mainly divided into two categories: the first category is based on the sub-module with fault self-clearing capability and the DC fast switch^6,7^; the second category is based on half-bridge sub-module without fault self-clearing capability and DCCB^8^. For the first kind of recovery scheme, after the line fault, the converter station needs to be locked to complete the restart of the system, which will cause a long time power interruption, have a great impact on the transmission system, and it is difficult to extend to the existing flexible DC system based on the half bridge sub-module. As for the second type of recovery scheme, the DCCB has the reclosing function, which has the advantages of fast recovery after fault and does not cause global locking. Therefore, this paper studies the reclosing strategy of DCCB for the second type of recovery scheme.

DC transmission lines are mostly overhead lines, with high probability of fault and most of them are transient faults. After fault disappearing, timely reclosing of DCCB can shorten the outage time of DC line, which can improve the power supply reliability of DC power grid. In the practical project, the DC circuit breaker adopts the method of automatic reclosing to restore power supply^9–11^. The specific realization method is: after the DCCB trips, the DC circuit breaker on one side of the DC line will automatically reclose after the de-ionization time of 200–300 ms. If the DC voltage of the fault line is established within a certain period, the DCCB on the opposite side will be reclosed, otherwise, the DCCB will be tripped again. In the above scheme, the DCCB will be reclosed regardless of the transient or permanent fault of the line. If the DCCB is closed to the permanent fault, it will cause a secondary impact on the entire transmission system^12,13^. Therefore, adaptive reclosing method is needed to distinguish the existence of line fault, so as to avoid secondary impact caused by incorrect reclosing and speed up the time for the system to restore power supply.

In view of the shortcomings of automatic reclosing schemes, domestic and foreign scholars have studied a series of adaptive reclosing schemes. In the literature^14^, it was suggested that once the fault current reached zero, the full-bridge MMC was employed to inject a current signal directly into the fault pole line, and the time of fault disappearance was determined by analyzing the response of the port voltage. However, injecting energy into the line before the fault disappears may prolong the fault extinction time. Literature^15^ proposed a scheme to realize fault protection and reclosure of DC power grid by changing the topology of the converter station sub-module of DC power grid, but the economics and engineering practicability of this scheme need to be further discussed. Literature^16^ used full-bridge MMC to generate pulse signals directly injected into the healthy pole line, and identified the fault nature by detecting the capacitive coupling signal of the fault pole line. This method needs to use the healthy pole injection signa. Literature^17^ proposed a cooperative control method between half-bridge MMC and hybrid DCCB, which used MMC to generate pulse signals to inject the signals directly into the fault pole line by unlocking hybrid DCCB in a short time. This method needs to change the existing MMC control strategy, and is difficult to apply to multi-terminal flexible DC network. Literature^18^ proposed an arc extinction time identification strategy based on the main frequency of line residual voltage for bipolar short circuit fault, but this strategy needs to be implemented in the frequency domain, with high computational complexity, and cannot be applied to single-pole ground fault. Literature^19^ used the difference of transition resistance before and after arc extinction to identify the arcing fault and confirm the arc extinguishing time, but this method is only for AC system, and the effectiveness of applying it to DC system needs to be further verified. Therefore, it is urgent to study the identification method of DC system line fault duration and fault arc extinguishing time.

Following a fault in a flexible DC grid, the residual voltage contains abundant fault characteristic information. When the fault arc extinguishes, significant changes occur in the amplitude and frequency characteristics of the residual voltage, which directly reflect whether the fault has been completely cleared. Analyzing from the essential perspective of physical principles, before the fault arc is extinguished, due to the fact that the fault point remains grounded all the time, the faulty pole cannot generate an induced voltage. Therefore, the residual voltage of the faulty pole will continuously oscillate around the zero axis and gradually decay until it finally drops to zero. After the fault arc is extinguished, an induced voltage will be generated on the faulty pole, thus giving rise to the typical phenomenon that before the fault disappears, the residual voltage oscillates and decays around the zero axis, and after the fault is completely eliminated, the residual voltage deviates from the zero axis. Moreover, the residual voltage can be directly measured and obtained by means of the existing voltage measurement equipment, without the need to additionally add other measurement devices. Compared with the schemes that achieve adaptive reclosing by injecting signals, the scheme of directly using the residual voltage to determine whether the fault arc is extinguished is simpler and easier to implement in actual operation, and has higher feasibility and operability. Based on these advantages, this paper pertinently proposes an adaptive reclosing scheme based on the S-transform of the residual voltage, aiming to provide more reliable technical guarantee for the safe and stable operation of the flexible DC power grid.

## Flexible DC grid topology

### Flexible DC grid topology with DCCB

**Fig. 1:**
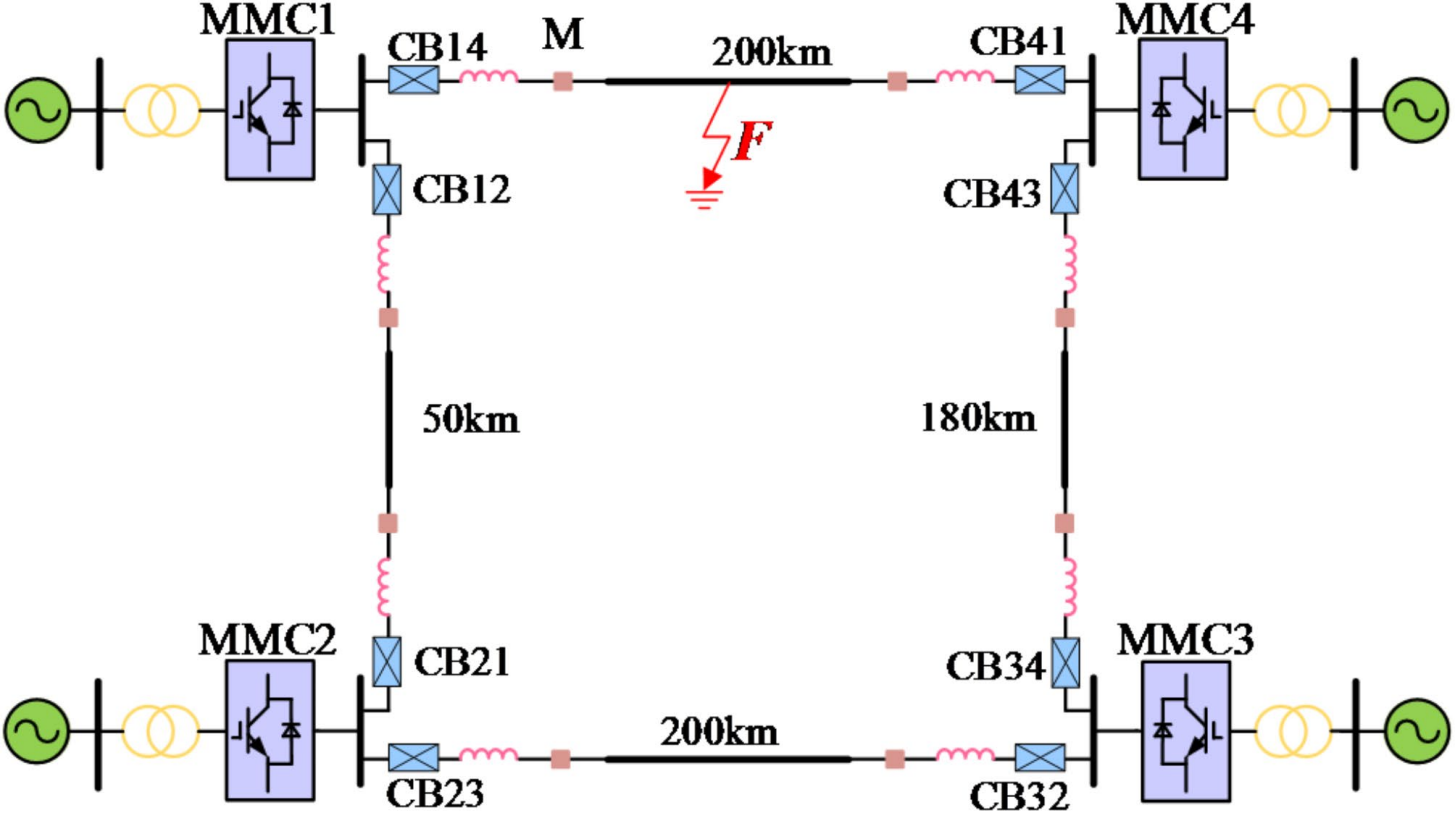
Flexible DC grid single line diagram.

The structure of the flexible DC grid configured with DCCB is shown in Fig. 1.

The DC voltage level is ± 500 kV. Both ends of the transmission line are equipped with smoothing reactors. The power grid adopts symmetrical double-pole grounding mode. Since the converter adopts a half-bridge sub-module structure without the ability of fault self-clearing, both sides of the line need to be equipped with high-voltage DCCBs for fault isolation.

### The topology and action sequence of hybrid DCCB

**Fig. 2:**
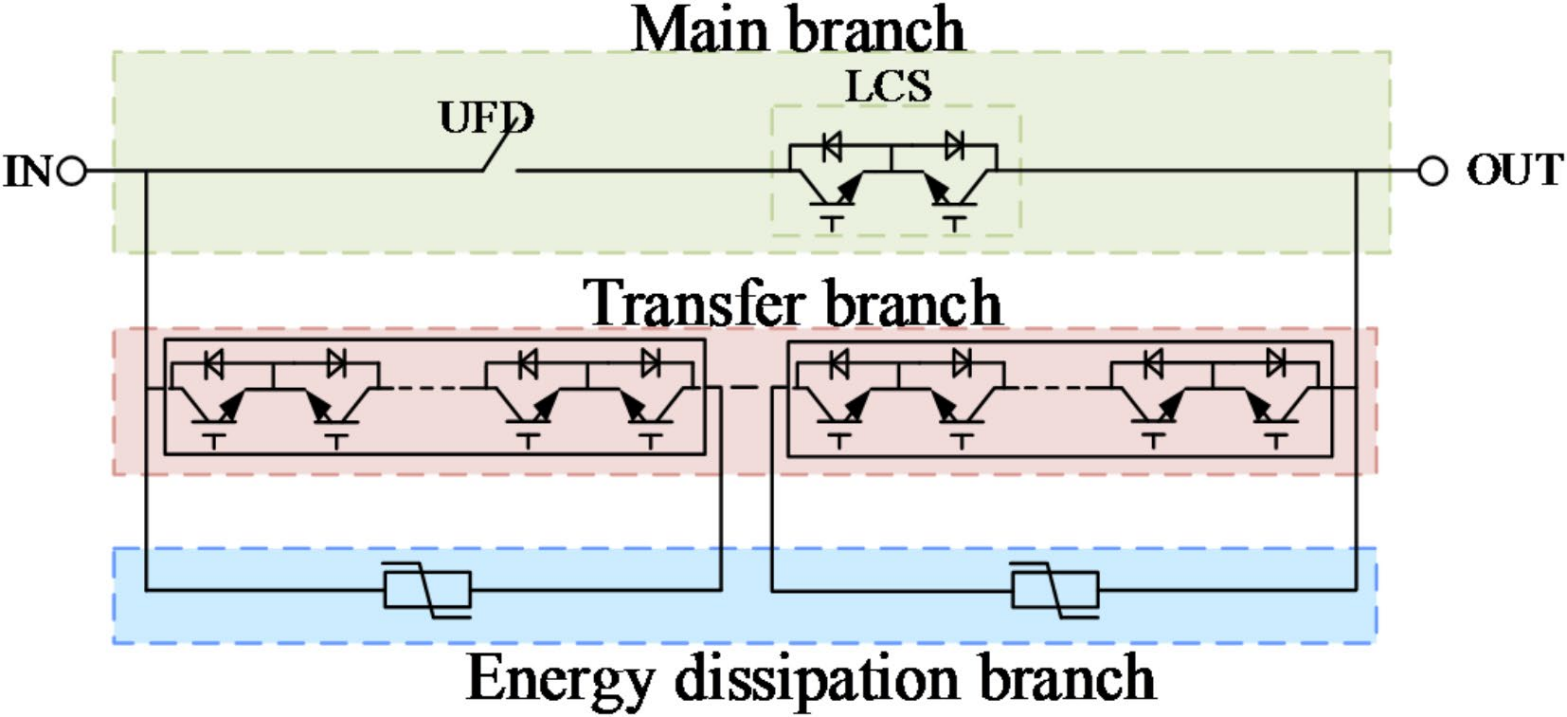
Hybrid DCCB topology.

The topology of hybrid DCCB is shown in Fig. 2, which mainly consists of main branch, transfer branch and energy-consuming branch connected in parallel^20^.

**Fig. 3:**
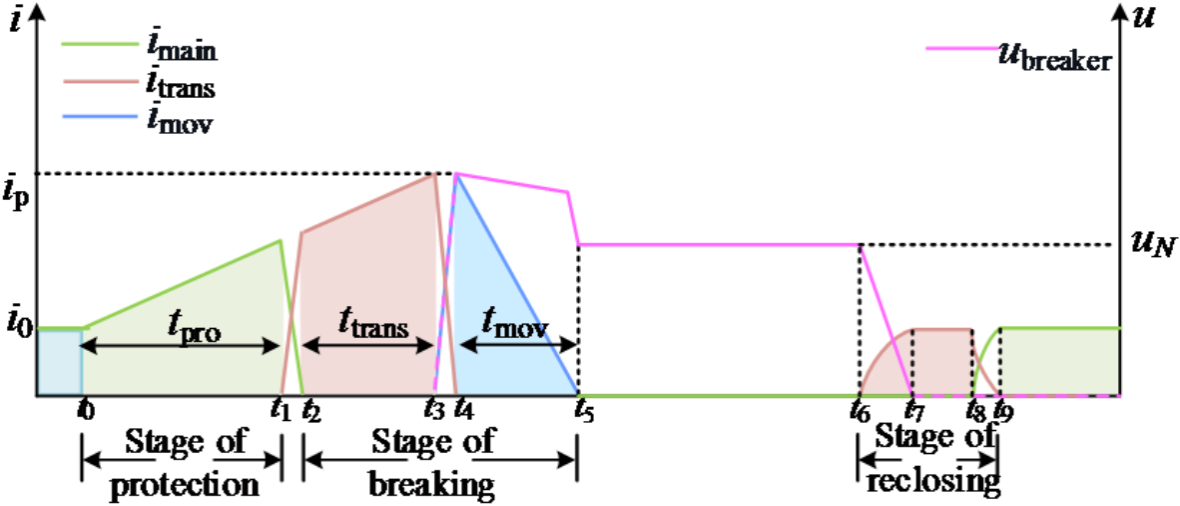
Schematic diagram of DCCB action sequence in case of transient fault.

In case of transient fault, the closing sequence of DCCB is shown in Fig. 3. At t_7_, the transfer branch current returns to the steady-state value, and the voltage at both ends of the DCCB is 0; t_7_–t_8_ is the mechanical switch closing stage; At t_8_, the mechanical switch is closed successfully; At t_9_, the current is completely transferred to the main branch, and the DCCB is successfully closed.

**Fig. 4:**
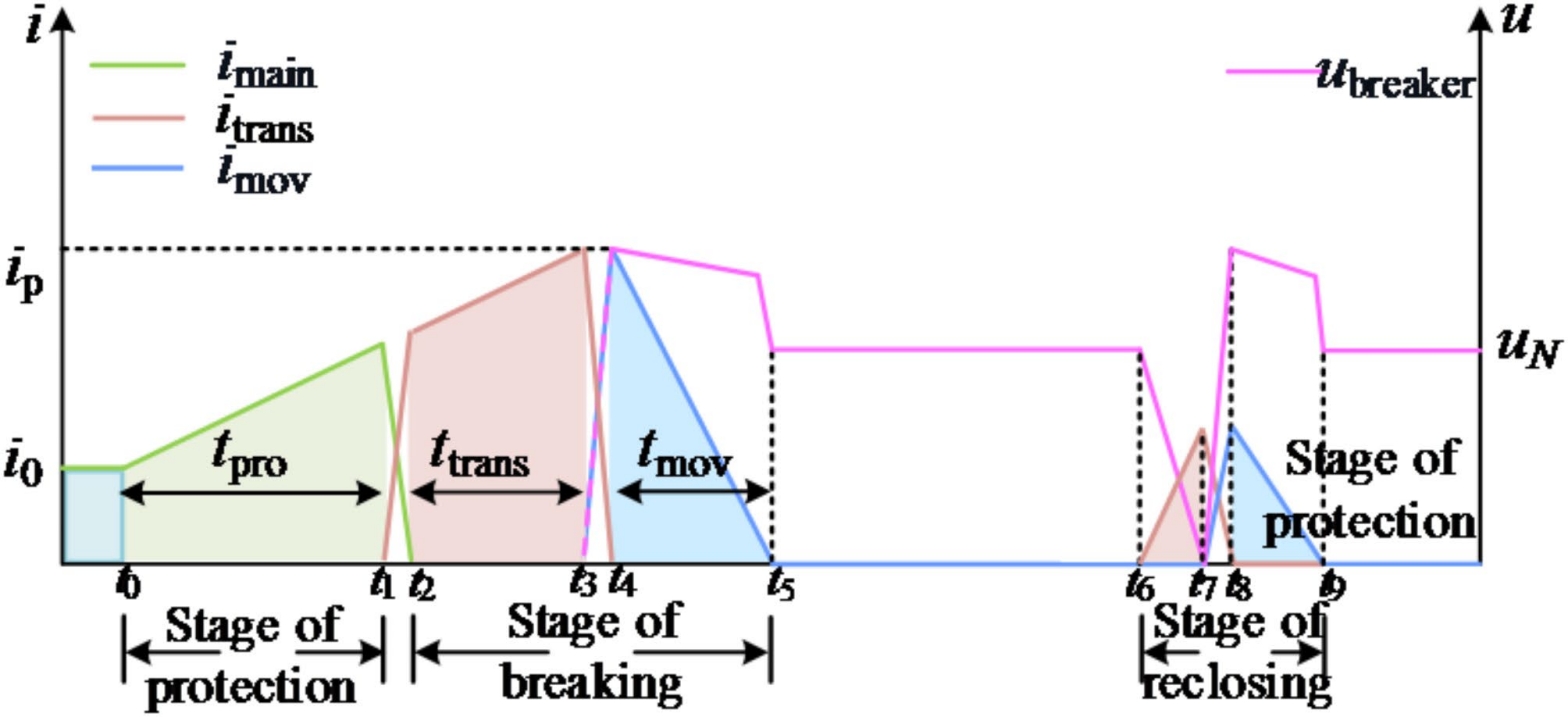
Schematic diagram of DCCB action sequence in case of permanent fault.

If a permanent fault occurs, the closing sequence is shown in Fig. 4. At t_7_, when the current of the transfer branch exceeds the protection threshold, the current is converted to the energy-consuming branch, and the voltage at both ends of the DCCB increases; At t_8_, the transfer branch voltage reaches the starting value of the arrester, and the current begins to decrease, and the voltage at both ends of the DCCB decreases; t_8_–t_9_ is the energy dissipation branch drainage stage; At t_9_, the current of the energy-consuming branch drops to 0, the voltage drop at both ends of the DCCB is the rated voltage of the system, and the DCCB fails to close.

## Residual voltage analysis of DC line

### Induced voltage calculation of transmission line

In the paper, the calculation of induced voltage is combined with the ideas of simulation charge method and image method. The key lies in the calculation of the simulated charge quantity. The simulation charge method equivalently represents the freely charged surface of the charged body with a set of discrete simulated charges. If the potential generated by the simulated charges on the boundary of the original field satisfies the given boundary conditions, then the simulated charge quantity can be calculated, and the potential at any point in the field can be calculated accordingly. Set up the simulated charge equations:

1 $$P\tau ={U_0}$$

where, *P* is the potential coefficient matrix, *τ* is the simulated charge matrix, and *U*_0_ is the potential matrix.

**Fig. 5:**
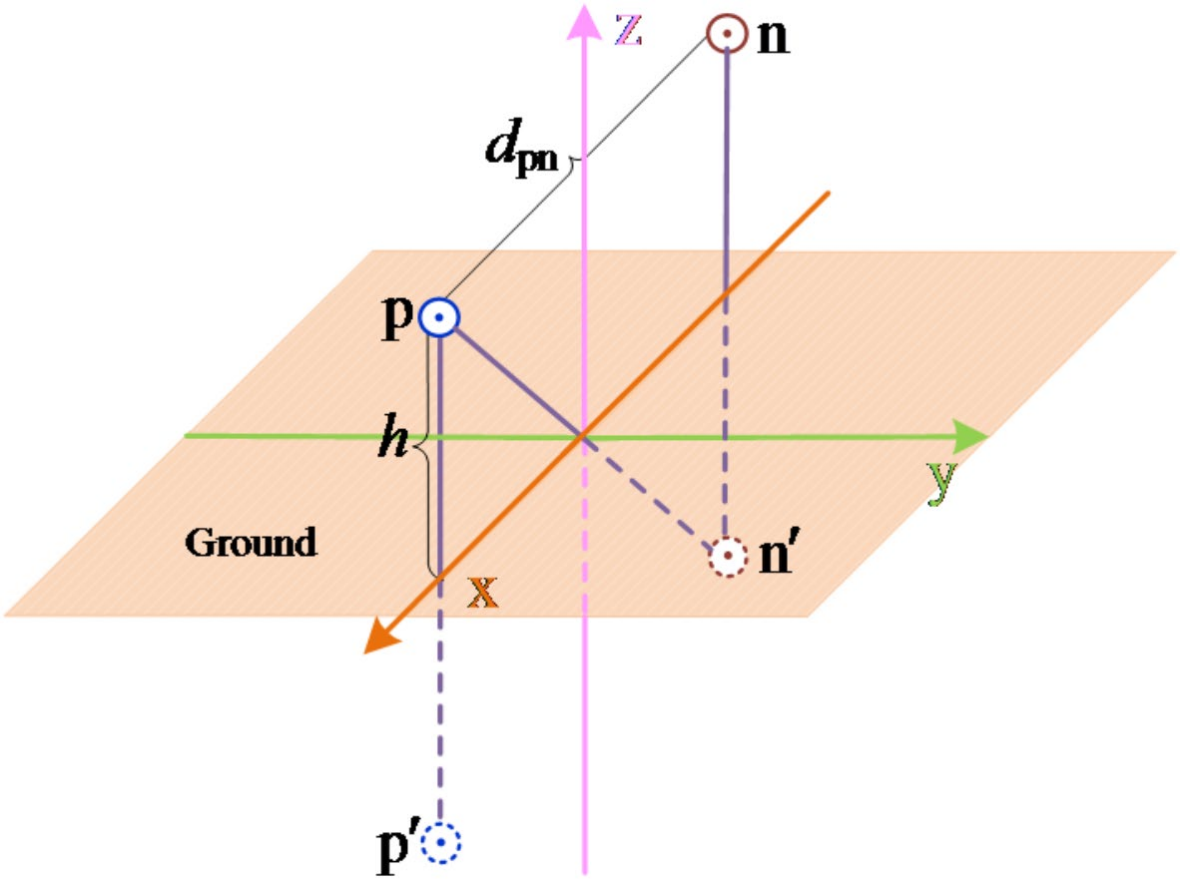
Calculation model of transmission line.

The ground is now set as an infinite ground plane with potential 0. The positive and negative transmission lines are equivalent to an infinite length of charge respectively. The calculation model of bipolar DC transmission line is shown in Fig. 5. In the figure, *p* and *n* are the positive line and negative line, *p*′ and *n*′ are the mirror image of the conductor, *R* is the equivalent radius of the split conductor, the positive and negative lines are horizontally erected, the horizontal distance between the conductors is *d*_*pn*_, the height to the ground is *h*, and the distance from the positive line *p* to the negative line *n* is *d*_*pn*_′.

The matrix equation can be listed according to (1):

2 $$\left[ {\begin{array}{*{20}c} {p_{{\text{pp} }} } & {p_{{\text{p} \text{n} }} } \\ {p_{{\text{n} \text{p} }} } & {p_{{\text{nn} }} } \\ \end{array} } \right]\left[ {\begin{array}{*{20}c} {\tau _{\text{p} } } \\ {\tau _{\text{n} } } \\ \end{array} } \right] = \left[ {\begin{array}{*{20}c} {u_{\text{p} } } \\ {u_{\text{n} } } \\ \end{array} } \right]$$

In the equation, *p*_*ij*_ represents the potential coefficient of the *j*-th line charge at the *i*-th line charge. When *i* = *j*, it is defined as the self-potential coefficient, and when *i* ≠ j, it is defined as the mutual potential coefficient. *τ*_*p*_ and *τ*_*n*_ are the simulated charges of the positive and negative line respectively, and *u*_*p*_ and *u*_*n*_ are the initial values of the potentials of the positive and negative lines, respectively. The formula for calculating the self-potential coefficient is as follows:

3 $${p_{ii}}=\frac{1}{{2\pi {\varepsilon _0}}}\ln \frac{{2h}}{R}$$

where, *ε*_0_ is the vacuum dielectric constant (generally taken as 8.85 × 10–12 F/m).

The calculation formula of mutual potential coefficient is:

4 $${p_{ij}}=\frac{1}{{2\pi {\varepsilon _0}}}\ln \frac{{d_{{ij}}^{\prime }}}{{{d_{ij}}}}$$

By combining (2), (3) and (4), it can be concluded that the simulated charge amount of the positive and negative pole lines are:

5 $$\tau _{{\text{p}}} = \frac{{p_{{\text{nn} }} u_{\text{p} } - p_{{\text{pn} }} u_{n} }}{{p_{{\text{pp} }} p_{{\text{nn} }} - p_{{\text{pn} }} p_{{np}} }}$$

6 $${\tau _{\text{n}}}=\frac{{{p_{{\text{pp}}}}{u_{\text{n}}} - {p_{np}}{u_p}}}{{{p_{\text{pp} }}{p_{\text{nn} }} - {p_{\text{pn} }}{p_{np}}}}$$

Since the distance between positive and negative poles is constant and the height to ground is equal, the self-potential coefficients of positive and negative pole are equal and the mutual potential coefficients are equal, then (5) and (6) can be simplified as follows, respectively

7 $$\tau _{{\text{p}}} = \frac{{p_{{\text{pp} }} u_{\text{p} } - p_{{\text{pn} }} u_{n} }}{{p_{{\text{pp} }}^{2} - p_{{\text{pn} }}^{2} }}$$

8 $${\tau _n}=\frac{{{p_{\text{pp} }}{u_n} - {p_{\text{pn} }}{u_p}}}{{p_{{\text{pp} }}^{2} - p_{{\text{pn} }}^{2}}}$$

In practical calculations, when applying the image method to equivalently represent the induced charge on the ground, a pair of line charges with the same magnitude but opposite signs are often formed. In this paper, the potential generated by the positive pole line charge *τ*_*p*_ and its mirrored charge *τ*_*p*’_ in the negative pole is:

9 $$\varphi =\frac{{{\tau _{\text{p}}}}}{{2\pi {\varepsilon _0}}}\ln \frac{{{d_{p^{\prime}n}}}}{{{d_{\text{pn} }}}}$$

where, *d*_*p*′*n*_ is the distance from the mirrored line *p*′ of the positive line to the negative line *n*, and *d*_*pn*_ is the horizontal distance between the positive and negative lines.

### Line fault characteristic analysis

#### Before the fault disappears

**Fig. 6:**
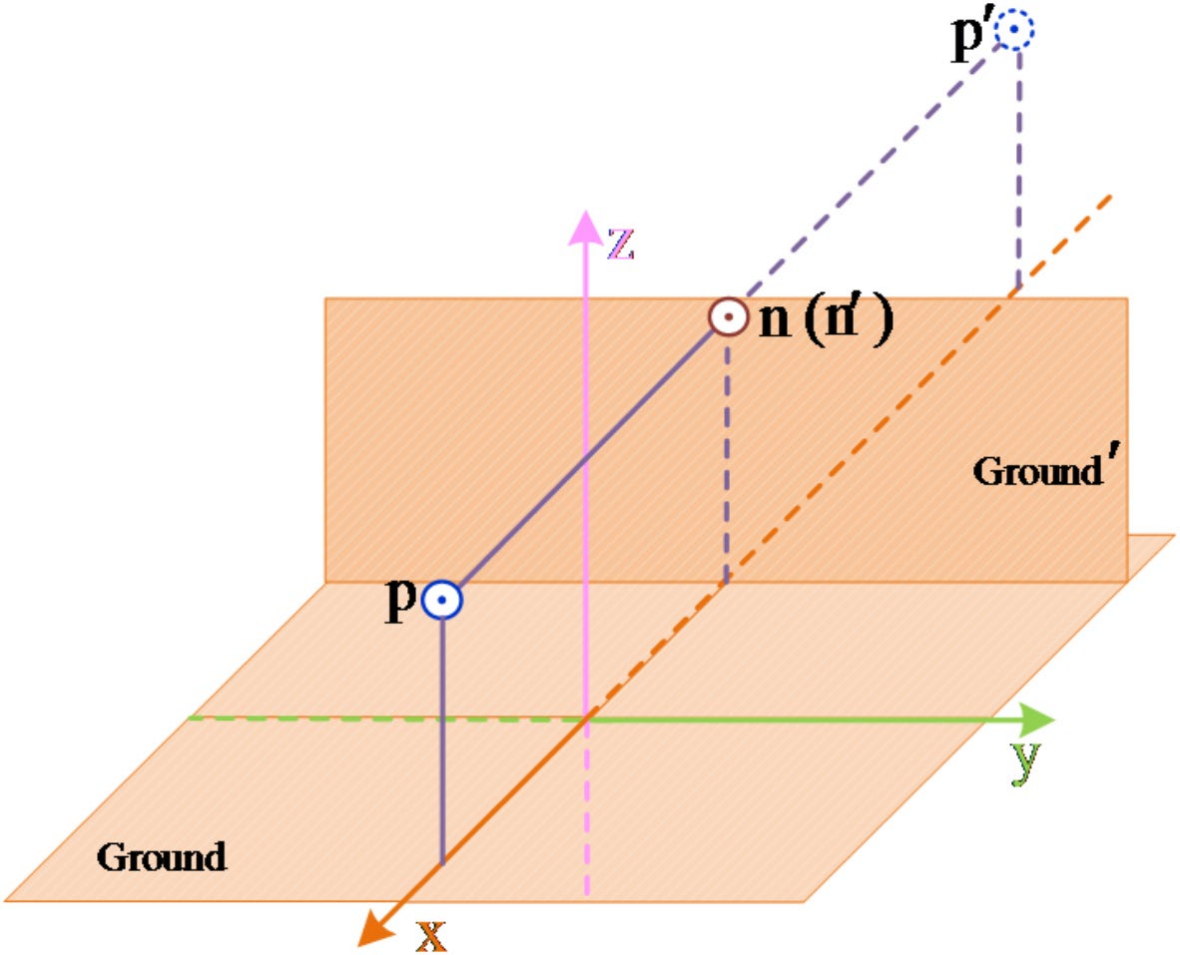
Calculation model of line before fault disappears.

Taking the metal fault of the negative line as an example, the calculation model of the transmission line before the fault extinguishes is shown in Fig. 6. The fault pole line is connected to the earth through the transition resistance. Since the DCCBs on both sides of the negative line have been disconnected, the potential of the negative line is the same as that of the earth.

The plane passing through the negative line and parallel to the *yoz* plane is selected as the potential reference plane. The potential generated by the positive pole line charge and its mirrored charge on the negative pole circuit is calculated according to (9). From the diagram, it can be seen that *d*_pn_=*d*_p′n_, so the potential at $\varphi =0$. Based on the above analysis, it can be concluded that if the fault does not extinguish, there is no induced voltage on the fault line.

According to the literature^21^, the energy stored in the electric and magnetic fields per unit length of the transmission line is

10 $${W_s}=\frac{1}{2}C{U^2}+\frac{1}{2}L{I^2}$$

The energy consumed per unit length of the transmission line is

11 $${W_c}=G{U^2}+R{I^2}$$

When the DCCBs on both sides of the fault line are disconnected, the energy stored in the inductance and capacitance is gradually consumed by the conductivity and resistance. Before fault extinguishing, the residual energy of the line will gradually decay to 0.

#### After the fault disappears

**Fig. 7:**
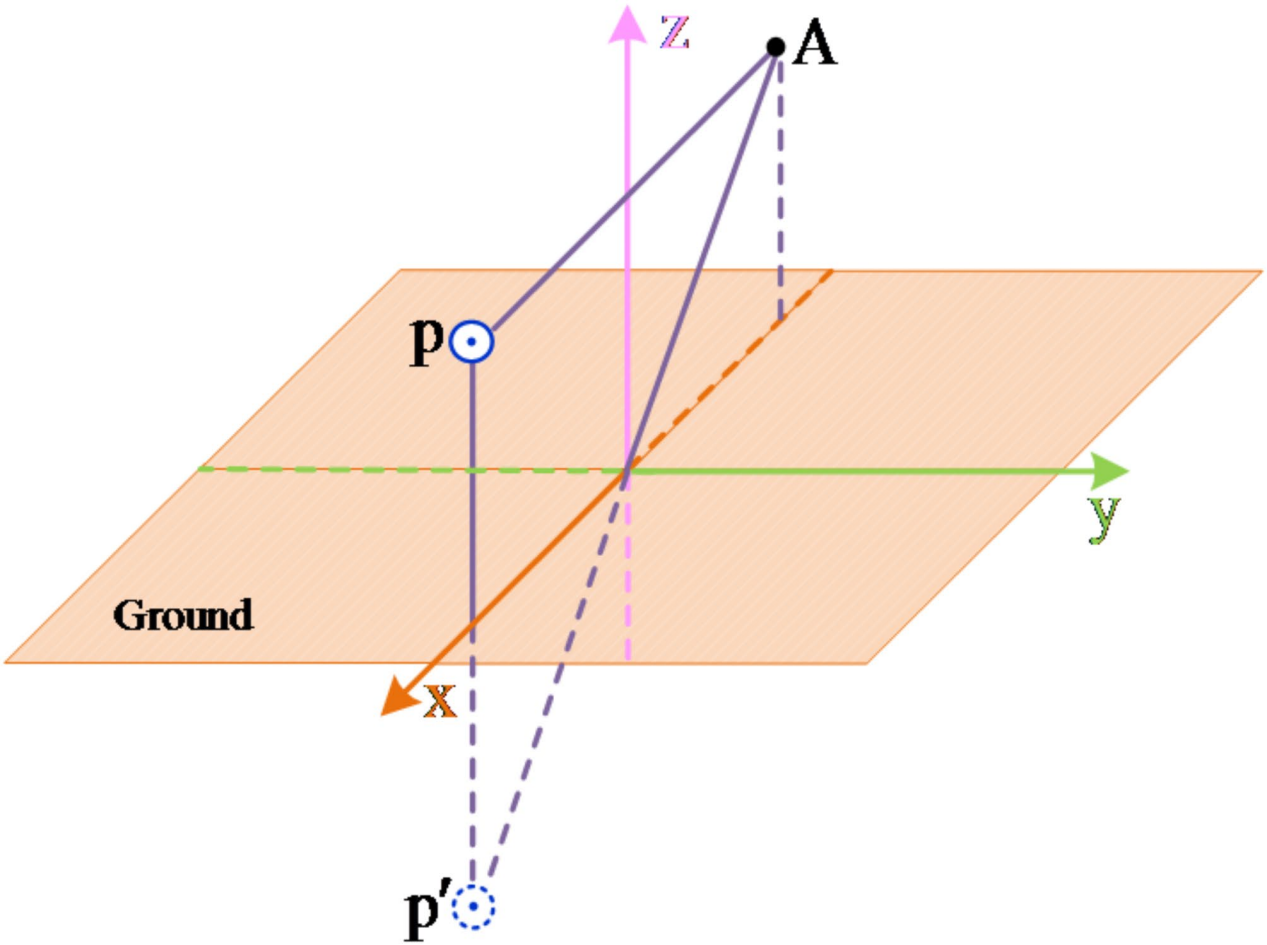
Calculation model of line after fault disappears.

Take the metal fault of the negative line as an example. The calculation model of the transmission line after the fault extinguishes is shown in the Fig. 7.

Select the *xoy* plane as the potential reference plane. Assuming that the field point A is a point on the negative line, the potential generated by the positive line charge and its image charge at A is

12 $$\varphi =\frac{{{\tau _{\text{p}}}}}{{2\pi {\varepsilon _0}}}\ln \frac{{{d_{p^{\prime}\text{A} }}}}{{{d_{\text{p} \text{A} }}}}$$

According to the above analysis, since the fault point is always grounded before the fault extinguishing, there is no induced voltage at the fault pole, so the residual voltage of the fault pole oscillates around the zero axis until zero, and there is induced voltage at the fault pole after the fault extinguishing, so that the residual voltage oscillates around the zero axis before the fault disappears, and the residual voltage shifts from the zero axis after the fault disappears.

**Fig. 8:**
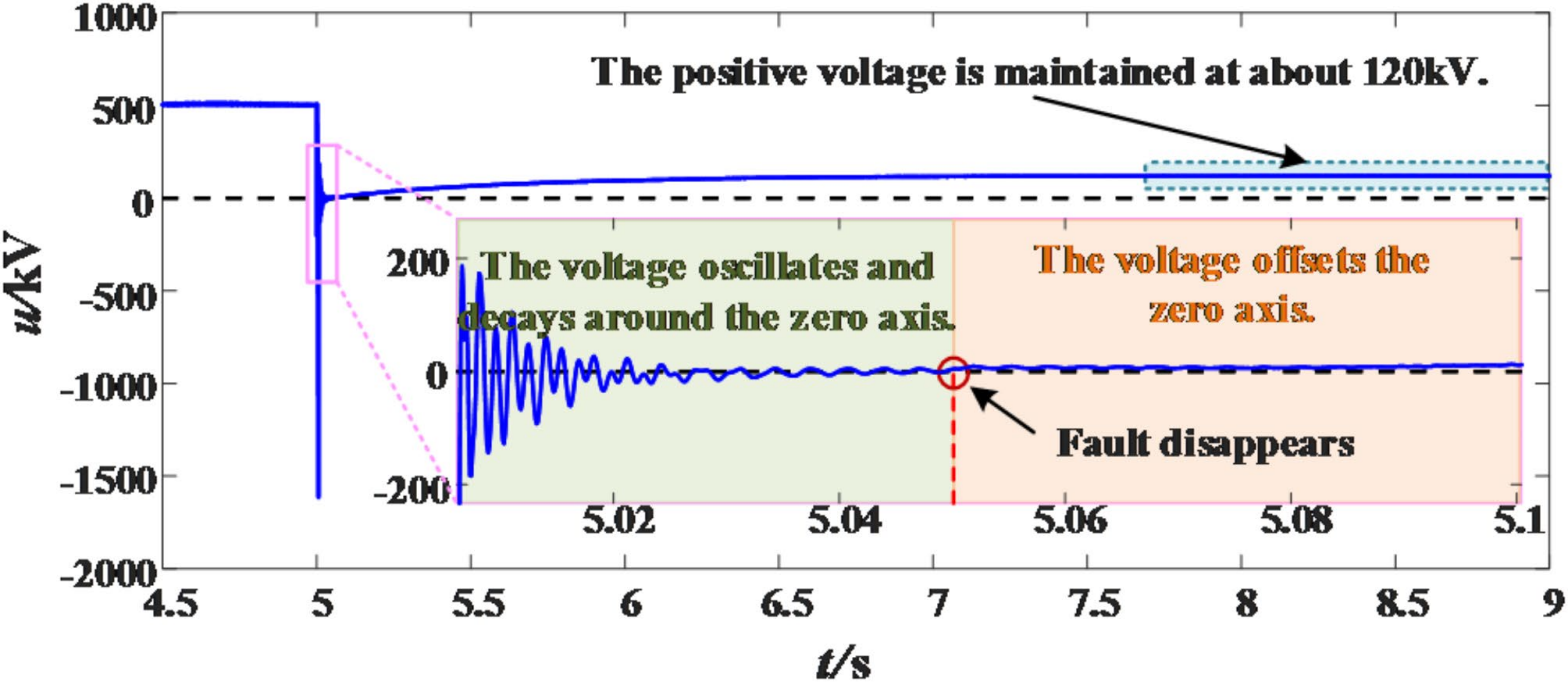
Voltage waveform at measuring terminal.

Taking the topology shown in Fig. 1 as an example, the equivalent radius of the pole line *R* = 0.162 m, the height of the pole line to the ground *h* = 30 m, and the distance between the poles *d*_*pn*_=16 m. It is assumed that a transient fault occurs on the positive line and the fault duration is 50 ms. According to (3) and (4), the self-potential coefficient and mutual potential coefficient are 1.0646 × 10^11^ and 2.4410 × 10^10^ respectively. The charge on the negative line calculated according to (8) is 4.9573 × 10^− 9^C. Then, according to (12), the induced voltage generated by the negative line at the positive pole through electrostatic induction is 121.0059 kV. In the PSCAD simulation model, the measured terminal voltage waveform of the above fault conditions is shown in the Fig. 8. It can be seen from the Fig. 8 that the measured terminal voltage oscillates and attenuates around the zero axis before the fault extinguishing, and after the fault extinguishing, the voltage gradually shifts and finally stabilizes at about 120 kV, which is close to the calculated value.

## Fault property identification method based on S transform

### S transformation method

S transform is a development of Fourier transform, which is usually used to analyze and process digital signals. The S-transform of signal *x*(*t*) is defined as follows:

13 $$S(\tau ,f) = \int_{{ - \infty }}^{\infty } {x(t)w(\tau - t,f)\text{e} ^{{ - {\text{j}}2\pi ft}} {\text{d}}t}$$

14 $$w(\tau - t,f) = \frac{{\left| f \right|}}{{\sqrt {2\pi } }}{\text{e}}^{{\left| {\frac{{ - f^{2} (\tau - t)^{2} }}{2}} \right|}}$$

where, *w*(*τ*−*t*, *f*) represents the window function of S-transformation; *f* represents the frequency.

The discrete representation of S-transformation is:

15 $$S\left[ {m,n} \right] = \sum\limits_{{k = 0}}^{{N - 1}} {X\left[ {n + k} \right]e^{{ - 2\pi ^{2} k^{2} /n^{2} }} e^{{{\text{j}}2\pi km/N}} } {\text{ }}n \ne 0$$

16 $$S\left[ {m,n} \right] = \frac{1}{N}\sum\limits_{{k = 0}}^{{N - 1}} {x\left[ k \right]} {\text{ }}n = 0$$

where.

17 $$X\left[ n \right] = \frac{1}{N}\sum\limits_{{k = 0}}^{{N - 1}} {x\left[ k \right]e^{{ - {\text{j}}2\pi kn/N}} }$$

In the S-transform modulus matrix, its column vector represents the distribution of signal amplitude with frequency at a certain time, and its row vector represents the distribution of signal amplitude with time at a certain frequency. The change of transmission line fault state can be expressed as the sudden change of voltage amplitude and frequency, which will inevitably be reflected in the module matrix obtained by S transformation of signal.

According to Parseval theorem, the energy in time domain is equal to the energy in frequency domain, and the energy will not change due to the transformation. Assuming that there is no spectrum leakage after S transformation, the average energy of the signal in a certain sampling interval can be expressed as

18 $$\frac{1}{T}\int {\left| {x\left( t \right)} \right|} ^{2} \text{d} t = \frac{1}{N}\sum\limits_{{k = 1}}^{N} {\left| {S\left( {\tau ,f_{k} } \right)} \right|^{2} }$$

The right side of the equation is the mean value of the sum of squares of the amplitudes (MSSA) of a column of the S matrix. In a period of time interval, the total energy of the signal is related to the change frequency and maximum amplitude of the signal.

**Fig. 9:**
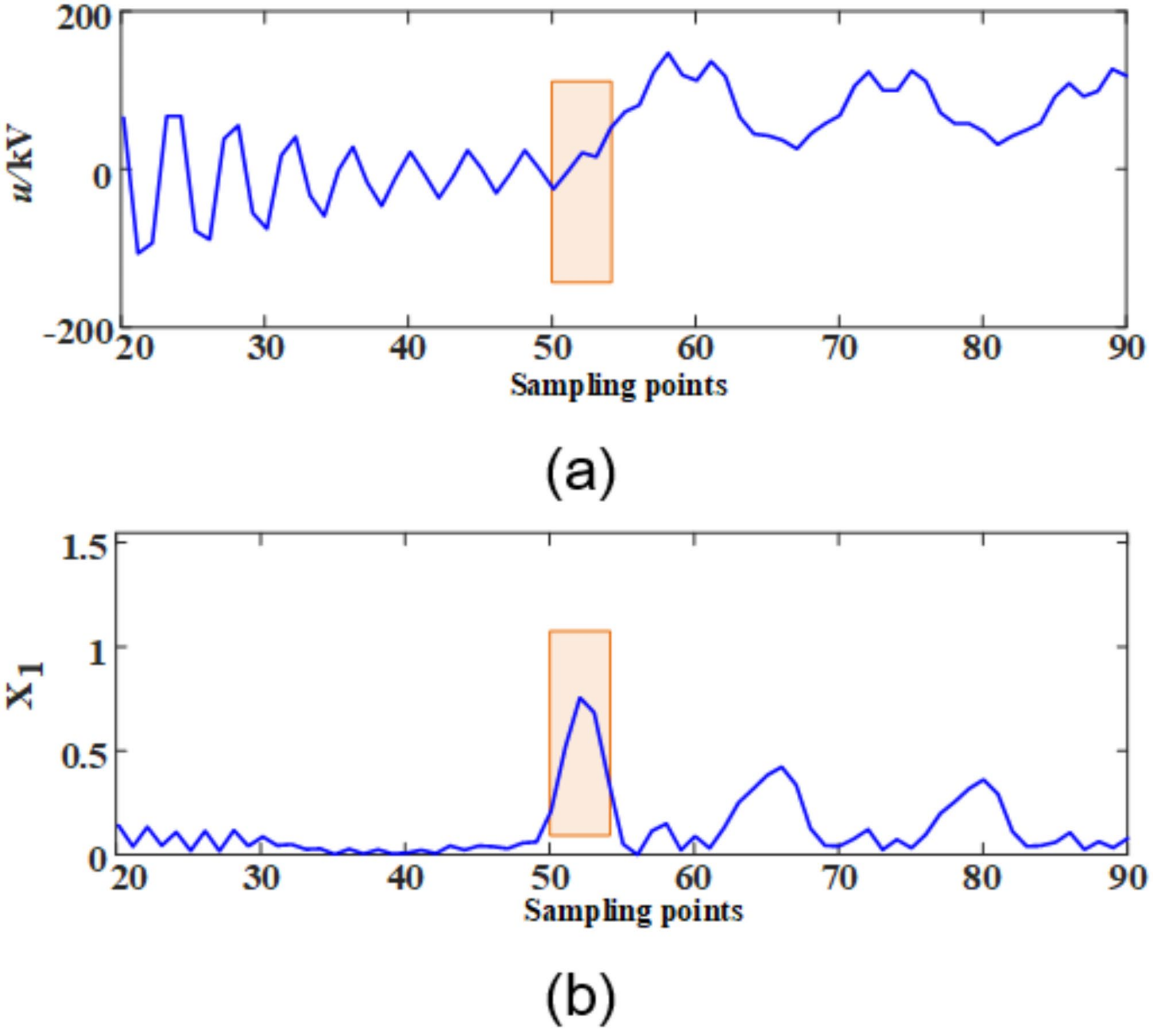
Comparison of waveforms before and after S-transform processing. (**a**) Initial waveform of fault voltage. (**b**) MSSA after S transformation.

In the power system, the abrupt changes in electrical fault signals are specifically manifested as significant variations in both the time domain and the frequency domain. After the fault signal is processed by the S-transform, the corresponding S-transform modulus matrix can be obtained. The MSSA of the S-transform matrix discussed in this paper comprehensively encompasses the abrupt change information in both the time domain and the frequency domain. To validate its effectiveness, a comparative analysis was conducted between the original voltage waveform and the amplitude mean square sum after S-transform processing, with the results shown in Fig. 9.

As clearly illustrated in Fig. 9, compared with the original fault waveform, the MSSA after S-transform processing demonstrates more pronounced transient characteristics. This result indicates that the S-transform method adopted in this paper can effectively enhance the transient magnitude of residual voltage when the fault disappears, thereby providing more reliable characteristic information for fault detection and analysis.

### Adaptive reclosing scheme based on S transform

**Fig. 10:**
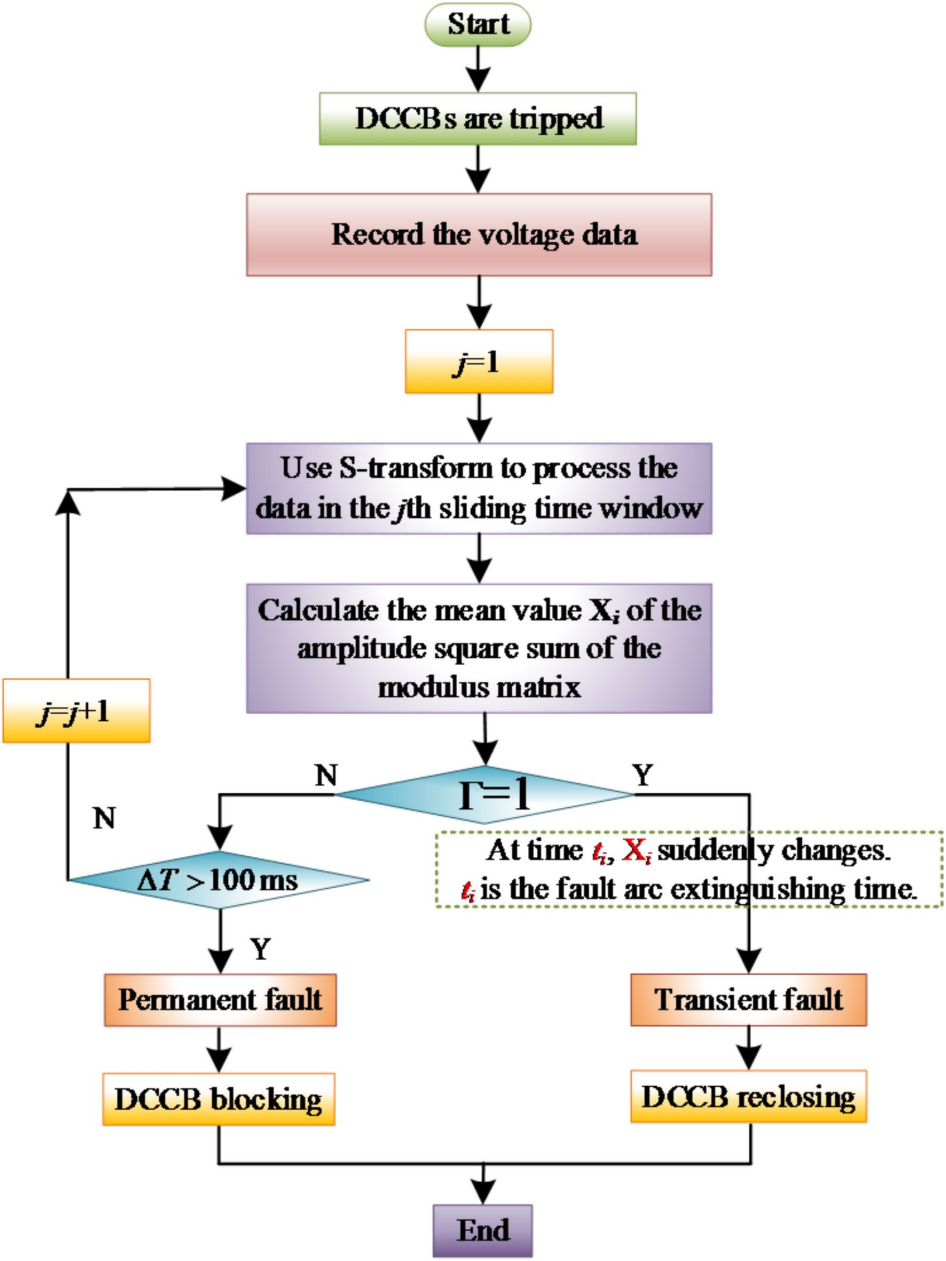
Flow chart of adaptive reclosing scheme.

Theoretically, in the detection time window, if the state of the transmission line does not change, the MSSA in the time window of this section will remain a constant value. If the state of the transmission line changes, the MSSA will change at the time of the state change. Therefore, judge whether the fault is extinguished by judging whether the MSSA changes in the detection window. Based on this, this paper proposes an adaptive reclosing scheme for flexible DC grid based on S transform. The scheme flow chart is shown in the Fig. 10.

The specific steps are as follows:

1. Protection device action after a line fault, DCCBs tripping, and the data acquisition device at the measuring end continuously records the fault data;
2. S-transform the fault voltage signal within a certain time window length at the measuring end;
3. Calculate the MSSA X_*i*_ of each column of the modulus matrix after S-transformation;
4. Judge whether there is a sudden change in the MSSA in the detection window, if so, the fault is judged to be extinguished in the window at that detection time, and the moment corresponding to the maximum sudden change point is the fault extinguished moment. Then after 100ms of deionization time, reclose the DCCBs. If not, repeat steps (2)–(4). Within the maximum detection time $\Delta T$, if the maximum mutation point of the MSSA in all sliding time windows does not exceed the threshold, the fault will be judged as permanent fault, and the DCCBs shall be locked immediately.

To express the above criterion visually, “Γ = 1” is specified for “X_*i*_ has mutation in the detection window”, and “Γ = 0” is specified for “X_*i*_ has not mutation in the detection window”.

### Time coordination between protection and adaptive reclosing

**Fig. 11:**
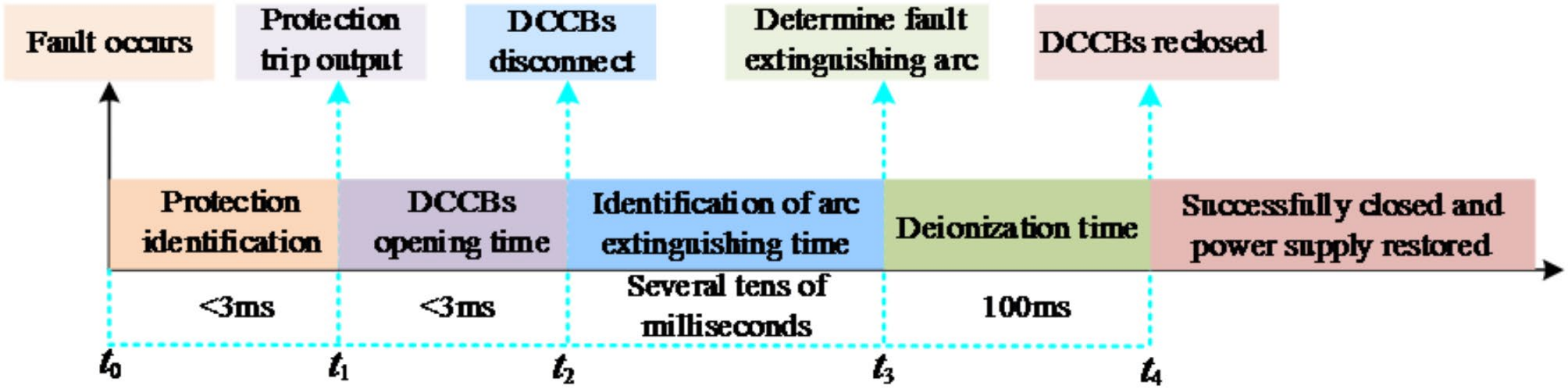
Time coordination between protection and adaptive reclosing.

In the flexible DC power grid, the time sequence coordination relationship between the protection scheme and the adaptive reclosing strategy is shown in the Fig. 11.

Once a fault occurs, the flexible DC power grid stipulates that the total time consumed for fault isolation and clearance generally should not exceed 6 ms. Considering that hybrid DCCBs usually require 3 ms for operation, the response time for fault isolation protection must be controlled within 3 ms. After the mechanical switch of the circuit breaker successfully completes the breaking action, the arrester is immediately put into operation and undertakes the task of energy dissipation. At the same time, the adaptive reclosing program is quickly activated to accurately identify the moment of fault arc extinction. After the arc is truly extinguished, although there is a short detection error, the state of fault arc extinction can still be detected in a timely manner. After that, the system keeps waiting until the insulation performance of the fault branch is completely restored, and then it will issue a reclosing command to drive the circuit breaker to perform the reclosing operation. If the reclosing operation of the circuit breaker is successful, the entire flexible DC power grid system can return to normal operation.

### Time window selection

#### Sliding time window

To analyze the real-time variation of the fault signal with time, a sliding time window with window length *h* and sliding factor *δ* is defined in *y*_*i*_(*t*).

19 $${\text{T}}(j,h,\delta ) = \{ y_{i} (t),j\delta \le t \le h + j\delta \} \quad j = 1,2 \ldots n$$

**Fig. 12:**
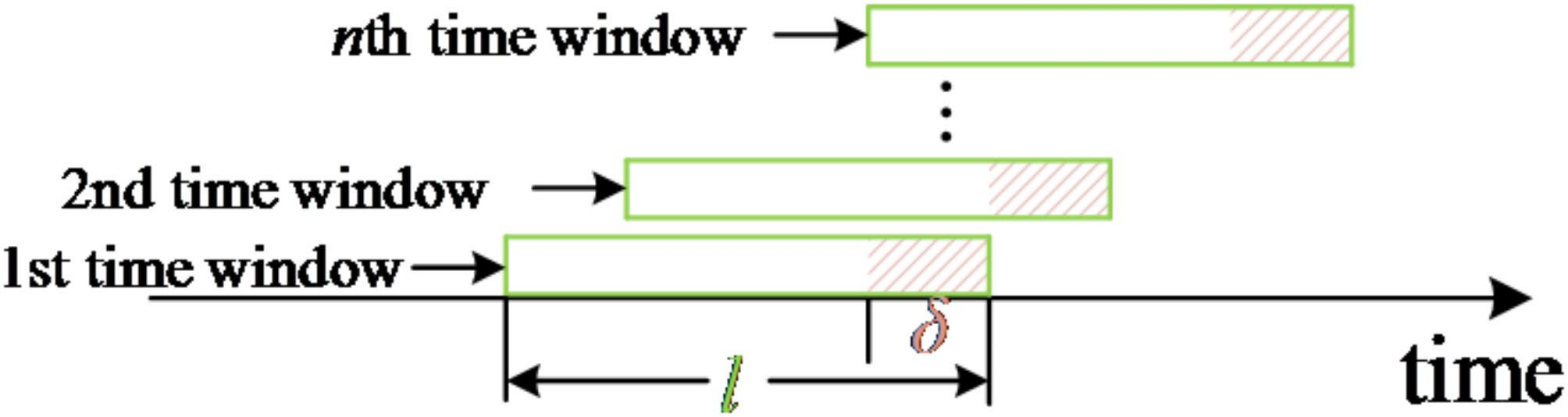
Sliding time window diagram.

The diagram of the sliding time window is shown in Fig. 12. After the analysis of the data in the 1st time window is completed, the data frame is slid forward according to the sliding factor *δ* to continue the next fault analysis.

In principle, the smaller the sliding time window length *h* and the sliding factor *δ*, the smaller the error of the detection result at the time of fault extinction time, but this will greatly increase the number of time window movements and calculation time, which is not conducive to the detection of the time of fault extinction time. In this paper, the sliding time window length is set to 10 ms and the sliding factor is set to 1 ms. This is merely presented as an example for elaboration and analysis. In practical specific application scenarios, relevant personnel can flexibly adjust and modify the values of the length of the sliding time window and the sliding factor according to the actual needs and specific situations.

#### Maximum detection time$\Delta T$

After fault extinguishing, it needs to wait for a period before reclosing. In the traditional automatic reclosing scheme, the fixed deionization time is 200–300 ms, which is taken as 200 ms in this paper. Since the time required for arc insulation recovery is 100 ms, the maximum detection time is 100 ms, that is, when the duration of transient fault is more than 100 ms, it will be determined as permanent fault and the DCCB will be locked.

## Simulation verification

To verify the reliability of the proposed method in this paper, a four-terminal flexible DC grid simulation model is built on the electromagnetic transient simulation software PSCAD/EMTDC platform, and the topology is shown in Fig. 1.

### Transient fault

**Fig. 13:**
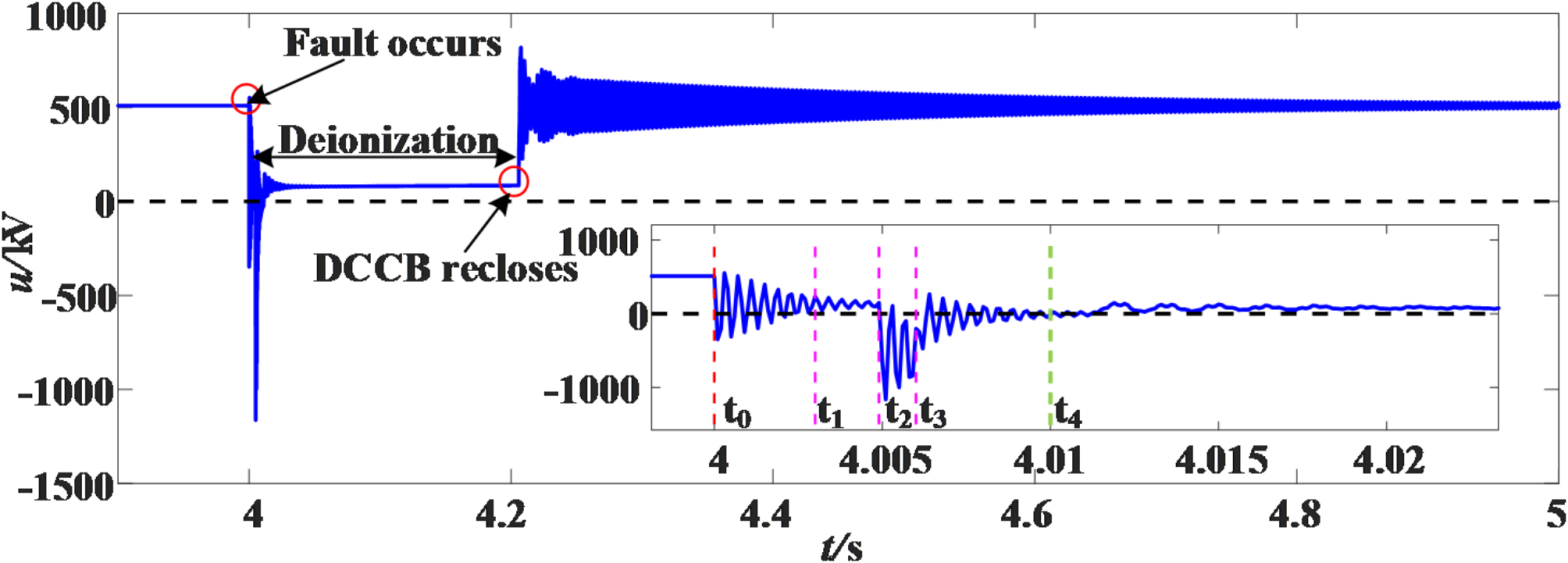
Transient fault voltage waveform.

It is now set that a transient grounding fault occurs at a distance of 20 km from the M measuring end of the positive line L_14_, with a duration of 10 ms and a sampling frequency of 10 kHz. The voltage waveform after the fault is as shown in the Fig. 13. In the enlarged figure, t_0_ is the time of the fault; t_1_ is the action time of the fast mechanical switch of the main branch in the DCCB, and the action duration is about 2 ms; t_2_ is the energy release time of the energy-consuming branch in the DCCB, and the energy release duration is about 1 ms; t_3_ is the completion time of DCCB action; t_4_ is the time when the fault disappears.

**Fig. 14:**
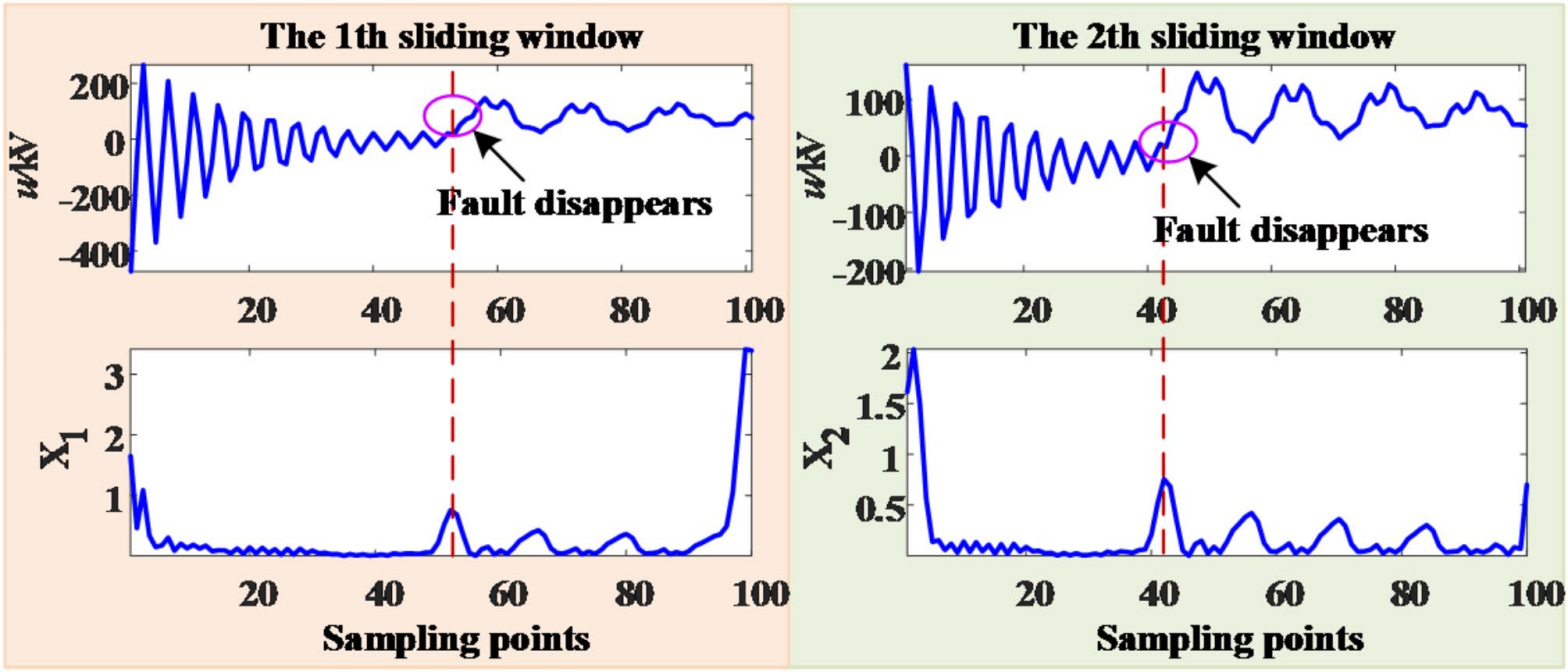
MSSA-time curve.

After the DCCBs are disconnected, the fault voltage signal at the measurement end is S-transformed, and calculate the MSSA of the elements in the module matrix according to the column. The MSSA-time curve is shown in the Fig. 14.

From the MSSA-time curve of the first sliding window, it can be seen that there is an obvious mutation at the 52nd sampling point, so it is judged that the fault will extinguish at the 52nd (5.2 ms) sampling point after the DCCBs are disconnected. Since the time of line protection and DCCB action after the fault is about 6 ms, the grounding fault will disappear at 11.2 ms from the start of the protection. In this simulation example, the duration of transient fault is 10 ms, with an error of only 1.2 ms. After fault extinguishing, it is necessary to wait for a period before reclosing operation. The purpose is to wait for the complete recovery of arc insulation. The recovery time of arc insulation is 100 ms^22^, so the DCCBs will reclose at 111.2 ms after the line fault.

**Fig. 15:**
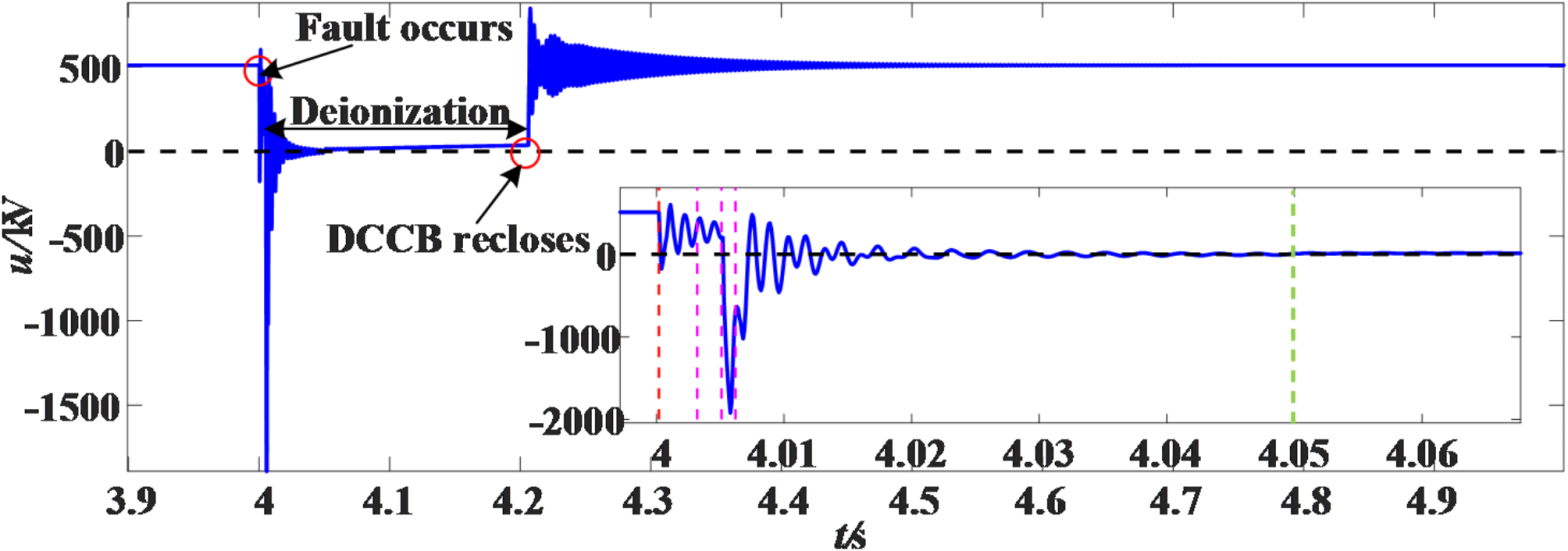
Transient fault voltage waveform.

It is now set that a transient grounding fault occurs at a distance of 100 km from the M measuring end of the positive line L_14_, with a duration of 50 ms and a sampling frequency of 10 kHz. The voltage waveform after the fault is as shown in the Fig. 15.

**Fig. 16:**
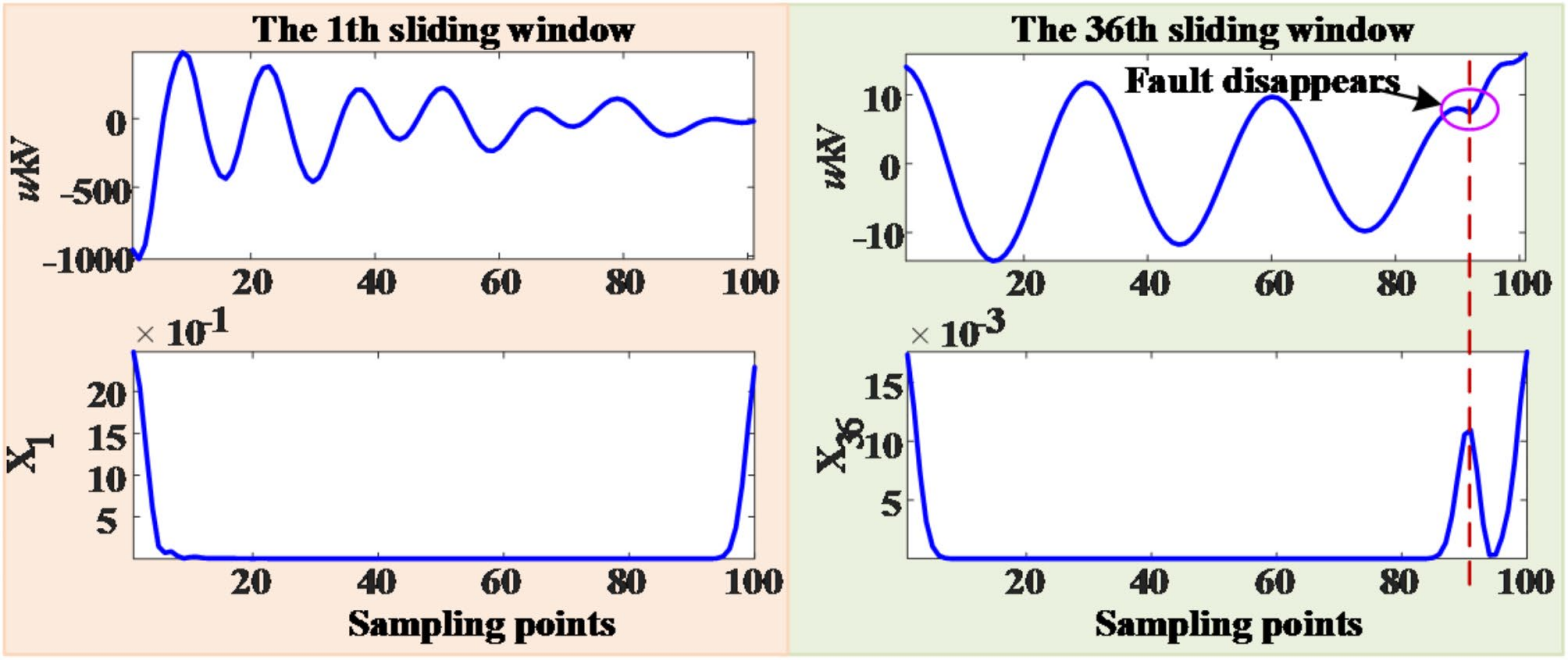
MSSA-time curve.

Since the fault duration is 50 ms, it is necessary to slide multiple time windows after the DCCBs are disconnected to detect the sudden change of the MSSA. Only the 1st time window after the DCCBs are disconnected and the time window when the fault disappears is detected are shown here. The MSSA-time curve in the above two time windows is shown in the Fig. 16.

As can be seen from the Fig. 16, the MSSA-time curve of the 1st sliding window remains constant, and there is a obvious abrupt change at the 92nd sampling point within the 36th time window, i.e. the sliding time window moves 35 times, so it is judged that the fault will extinguish at 44.2 ms after the DCCBs are disconnected. Since the action time of the line protection and DCCBs after the fault is about 6 ms, the ground fault is extinguished at 50.2 ms from the start of the protection. In this simulation example, the duration of the transient fault is 50 ms, with an error of only 0.2 ms. Considering the deionization time of 100 ms, the DCCBs will reclose at 150.2 ms after the fault.

Compared with the fixed 200–300 ms deionization time of traditional automatic reclosing, this method greatly improves the reclosing efficiency and is more conducive to the rapid restoration of power supply.

### Permanent fault

**Fig. 17:**
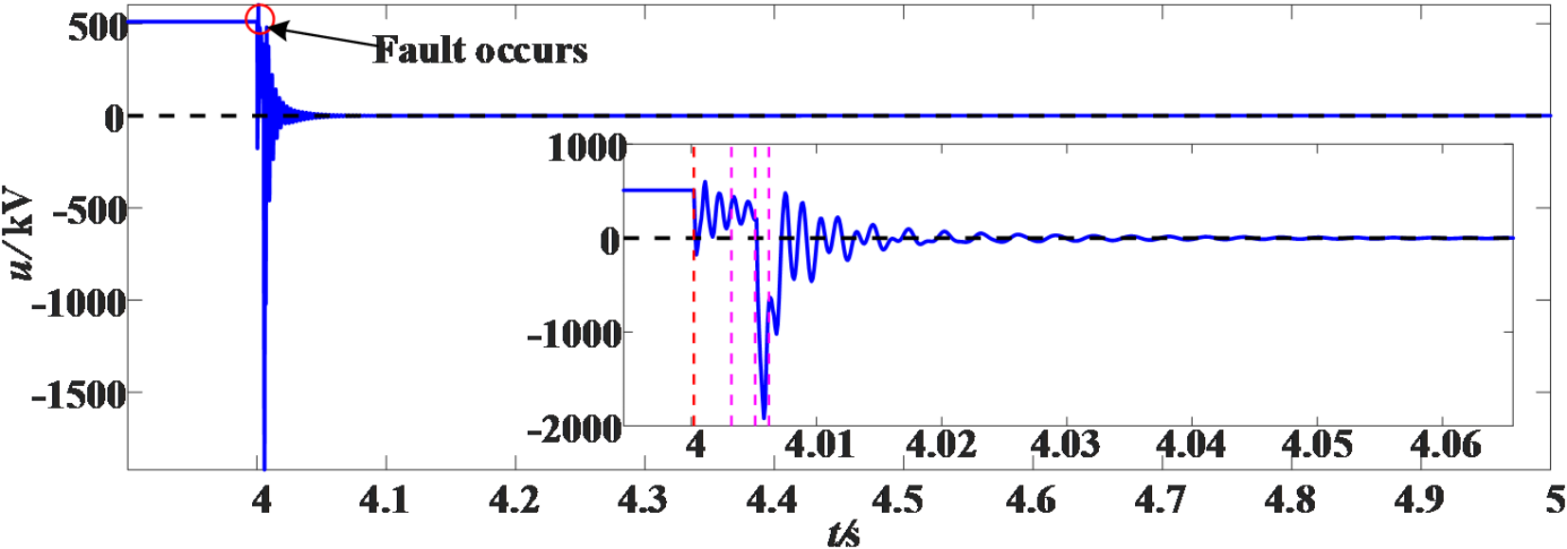
Permanent fault voltage waveform.

It is now assumed that permanent ground fault occurs at 100 km from the M measuring end of the positive line L_14_, and the voltage waveform after the fault is as shown in the Fig. 17.

**Fig. 18:**
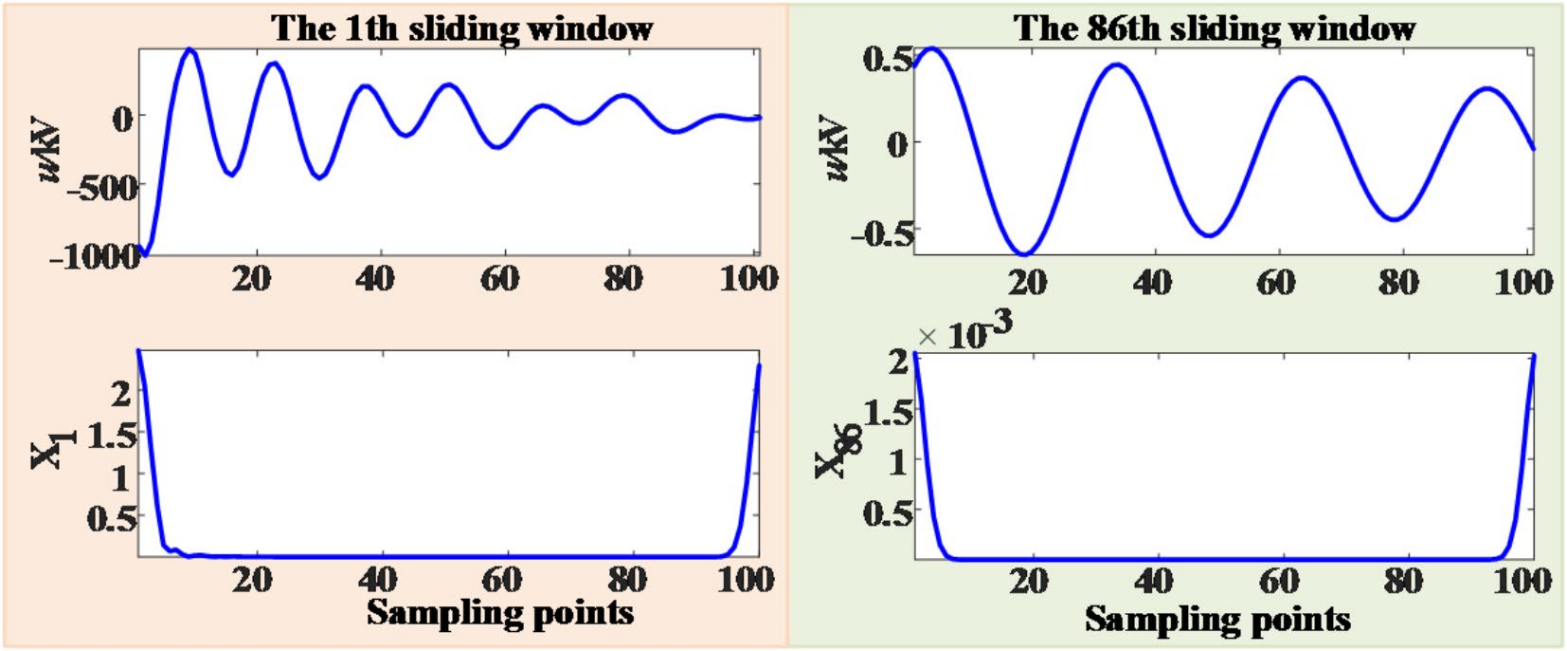
MSSA-time curve under permanent fault.

The length of detection time set in this paper is 100 ms after the fault, while the time of line protection and circuit breaker action is about 6 ms, so whether the fault exists is judged within 94 ms after the DCCBs are disconnected. Only the MSSA-time curve of the first time window and the last time window after the DCCBs are disconnected are shown here, as shown in the Fig. 18.

It can be seen from Fig. 18 that within the detection time, the MSSA-time curve in each sliding window have no sudden change, so the fault type is determined as permanent fault, and the DCCBs should be locked immediately.

### Adaptability analysis

#### Effect of fault resistance

To verify the influence of fault resistance on adaptive reclosing strategy, a single-pole grounding fault is set at 10 km away from the converter station on line L_14_. The fault resistance is 0.01 Ω, 50 Ω, 100 Ω and 200 Ω, respectively. The determination results of adaptive reclosing are shown in Table 1.

**Table 1:** Judgment results of different fault resistances.

Fault nature	Fault location/km	Fault resistance/Ω	Actual moment of fault disappearance/ms	Time window sequence number at Γ = 1	Detected fault disappearing moment/ms	Detection error/ms	Identification results
Transient fault	35 km	0.01	18	4	18.4	0.4	Transient fault
		50		4	18	0	Transient fault
		100		4	18.4	0.4	Transient fault
		200		5	18.6	0.6	Transient fault
Permanent fault	35 km	0.01	–	–	–	–	Permanent fault
		50		–	–	–	Permanent fault
		100		–	–	–	Permanent fault
		200		–	–	–	Permanent fault

It can be seen from the table that the method proposed in this paper is not affected by the fault resistance.

#### Effect of fault distance

In order to verify the effect of fault distance on the adaptive reclosing strategy, the positive metal grounding fault is set in line L_14_, which is located at 15 km, 40 km, 100 km, 160 km and 195 km from the converter station, and its determination results are shown in Table 2.

**Table 2:** Judgment results for different fault distance.

Fault nature	Fault resistance/Ω	Fault distance/km	Actual moment of fault disappearance/ms	Time window sequence number at Γ = 1	Detected fault disappearing moment/ms	Detection error/ms	Identification results
Transient fault	0.01	15	26 ms	14	27.6	1.6	Transient fault
		40		12	26.4	0.4	Transient fault
		100		13	26.4	0.4	Transient fault
		160		13	26.6	0.6	Transient fault
		195		14	27.8	1.8	Transient fault

#### Comparison of reclosing time optimization effects

**Fig. 19:**
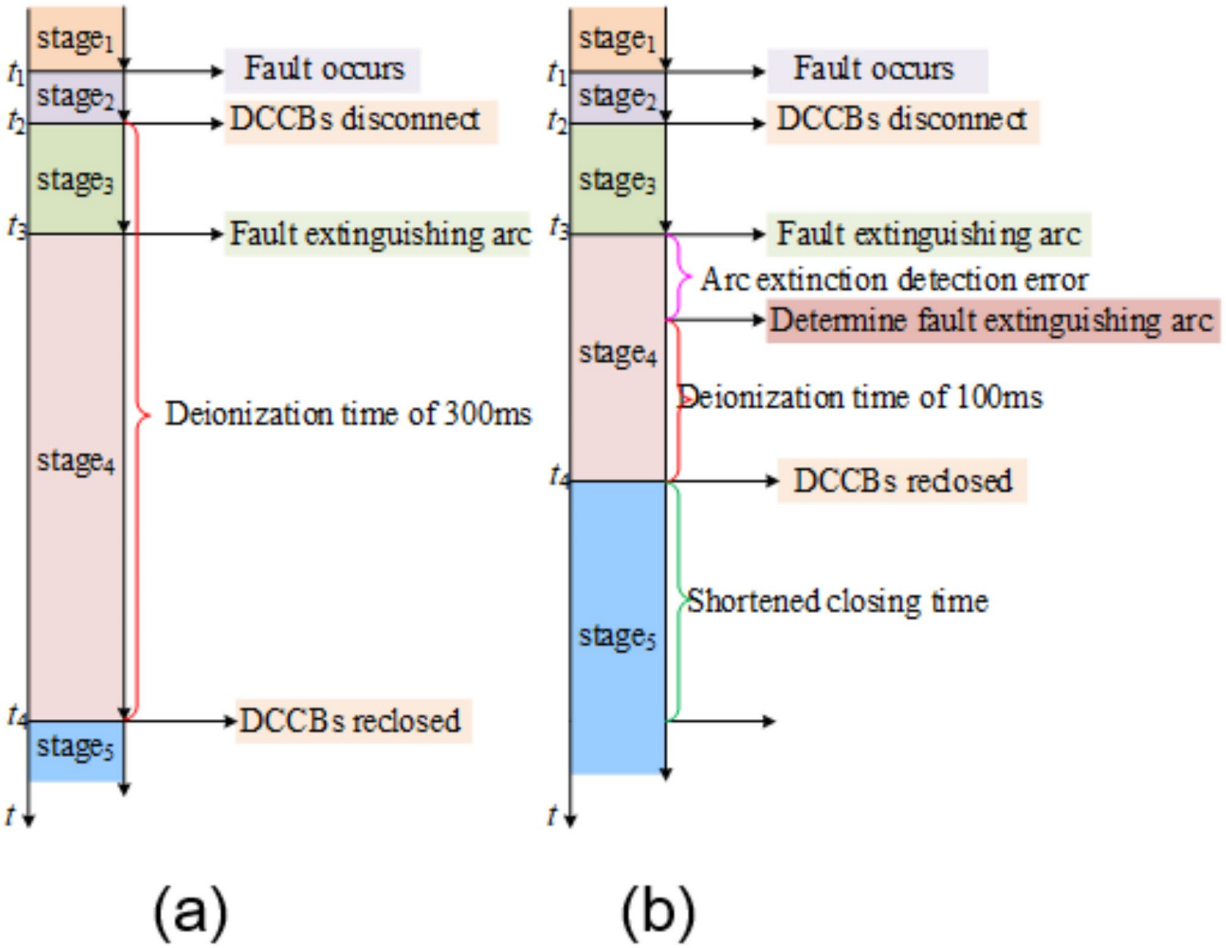
Comparison of different reclosing strategies. (**a**) The automatic reclosing (**b**) The adaptive reclosing.

A comparison of the timing between traditional automatic reclosing and the adaptive reclosing proposed in this paper is shown in Fig. 19.

For the traditional conventional automatic reclosing, after the circuit breaker trips, it is necessary to wait for a fixed deionization time of 200 ms before the reclosing operation can be carried out. In sharp contrast, the adaptive reclosing with the ability to determine the arc extinction moment can conduct real-time monitoring of whether the fault arc has been extinguished after the circuit breaker trips. Once it is accurately determined that the fault arc has been extinguished, only an additional deionization time of 100ms is required, and then the reclosing operation can be immediately carried out.

**Fig. 20:**
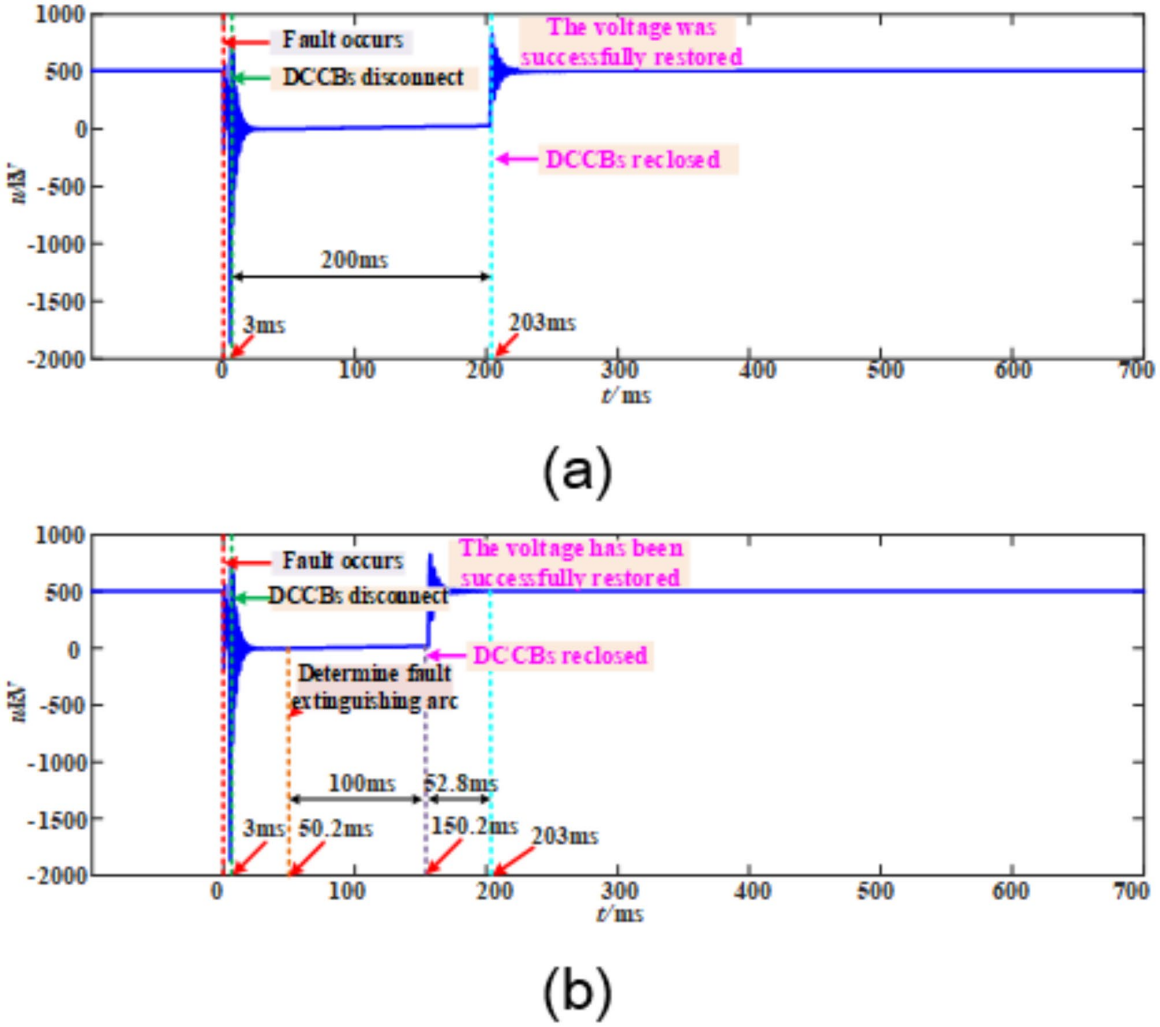
Transient fault.

Taking a transient fault occurring at 100 km from the M-end measurement point on the positive pole of line L14 as an example, the fault duration is 50 ms. Figure 20 shows the comparison of processing results between the automatic reclosing strategy and the proposed strategy. In the automatic reclosing strategy shown in Fig. 20a, after the circuit breaker trips, a fixed delay of 200 ms is required before reclosing can be performed. For transient faults, although the fault may have been cleared earlier, the system still needs to wait for the full 200 ms delay, which not only prolongs unnecessary power outage time but also significantly affects the timeliness of fault recovery. In contrast, in the adaptive reclosing strategy shown in Fig. 20b, after the circuit breaker trips, the system immediately initiates real-time arc extinction detection. Upon detecting arc extinction at 50.2 ms, the system only needs an additional 100 ms of insulation recovery time before issuing the reclosing command, ultimately achieving successful voltage restoration. Compared with the automatic reclosing strategy, the proposed method can advance power supply restoration by 52.8 ms, significantly improving fault recovery efficiency.

**Fig. 21:**
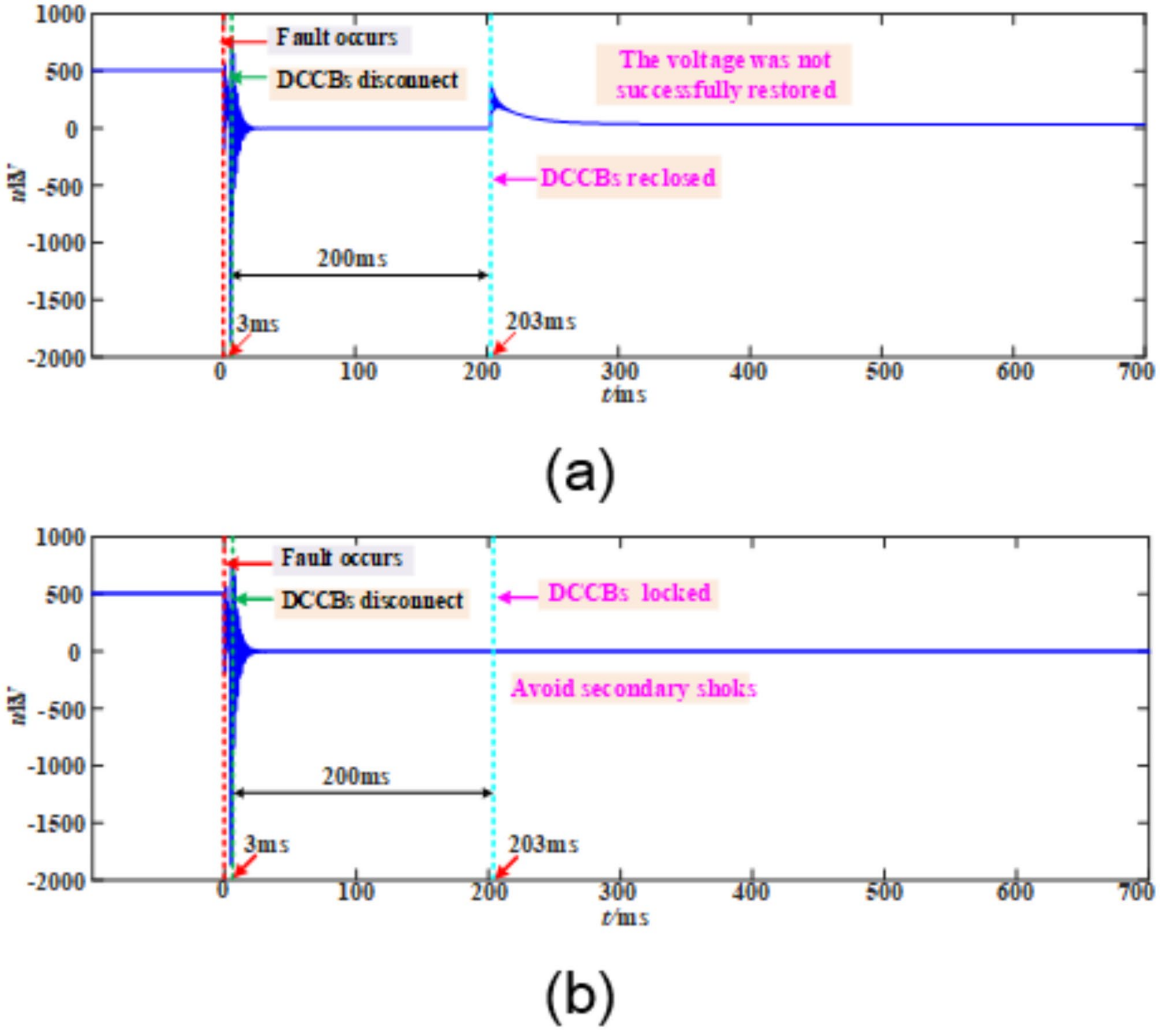
Permanent fault. (**a**) The automatic reclosing strategy. (**b**) The adaptive reclosing strategy proposed in this paper.

Taking a permanent fault occurring at 100 km from the M-end measurement point on the positive pole of line L14 as an example, Fig. 21 shows the comparison of processing results between the automatic reclosing strategy and the proposed strategy. In the automatic reclosing strategy shown in Fig. 21a, after the circuit breaker trips, a fixed delay of 200 ms is required before reclosing is performed. Since the fault persists, the system voltage fails to establish, and this reclosing on a permanent fault causes a secondary impact on the system, seriously threatening its safe and stable operation. In contrast, in the adaptive reclosing strategy shown in Fig. 21b, the system continuously detects the presence of the fault throughout the detection process and accurately identifies permanent fault characteristics at the end of the deionization period, thereby executing reclosing blocking. This method effectively avoids secondary impacts caused by reclosing on permanent faults, ensuring the safe and stable operation of the system.

As shown in Fig. 20, when a transient fault occurs on the line, compared with the traditional automatic reclosing strategy, the proposed adaptive reclosing strategy can significantly reduce power supply restoration time, achieving faster system recovery. The results in Fig. 21 demonstrate that when a permanent fault occurs on the line, the proposed adaptive reclosing strategy can reliably block circuit breaker operation, effectively avoiding secondary impact damage to the system caused by reclosing on permanent faults, thereby ensuring safe and stable system operation.

#### Comparison with existing adaptive reclosing strategies

Currently, adaptive reclosing methods are primarily implemented based on the following four principles: traveling wave principle, line residual voltage principle, DC circuit breaker topology principle, and signal injection principle. To fully demonstrate the superiority of the proposed adaptive reclosing scheme, we conducted a systematic qualitative comparison between the proposed method and existing approaches, with the specific comparative results presented in Table 3.

**Table 3:** Comparison of different methods.

	Based on traveling wave^18^	Based on sidual voltage of line^23^	Based on DCCB ^24^	Based on injection signal^25^	The proposed scheme
Detection of fault disappearing time	No	No	No	No	Yes
Real-time detection	Yes	No	No	No	Yes
Additional controls or devices	Yes	Yes	Yes	Yes	No

It can be clearly seen from the comparison results in the above table that the adaptive reclosing method proposed in this paper has the ability to accurately detect the moment of fault arc extinction. It is particularly worth mentioning that during the actual application of this method, there is no need to additionally install complex equipment, nor is it necessary to make any changes to the existing control mode of the converter station, demonstrating extremely high practicality and convenience.

In addition, in order to more vividly demonstrate the remarkable advantages of the method proposed in this paper in reliably detecting the moment of fault arc extinction, a rigorous quantitative comparative analysis is carried out between this method and the method proposed in Reference^26^. Through a comprehensive and meticulous comparison, the differences between the two are presented with accurate data. The specific comparison results are shown in the following Table 4.

**Table 4:** Comparison of methods.

Fault location	Fault resistance/Ω	Actual moment of fault disappearance/ms	Method proposed in literature^26^			The method proposed in this paper		
			Detected moment of fault disappearance/ms	Detection error/ms	Identification results	Detected moment of fault disappearance/ms	Detection error/ms	Identification results
Head end	0	19	24	5	Transient fault	19.3	0.3	Transient fault
	100	38	42	4		39.6	1.6	
	300	30	32	2		30.1	0.1	
Midpoint	0	29	30	1	Transient fault	29.4	0.4	Transient fault
	100	34	41	7		34.1	0.1	
	300	33	38	5		34.5	1.5	
Terminal	0	39	42	3	Transient fault	39.1	0.1	Transient fault
	100	30	37	7		30.5	0.5	
	300	34	35	1		35.2	1.2	

It can be clearly seen from the data in the Table that the method proposed in this paper demonstrates higher precision in detecting the moment of fault arc extinction. The error of the detected arc extinction moment is smaller compared with other methods. This advantage enables this method to significantly optimize the reclosing time for transient faults, achieve the rapid restoration of power supply.

## Conclusion

This paper analyzes the induced voltage before and after the fault extinction of the transmission line fault, proposes an adaptive reclosure scheme for the flexible and direct power grid based on S transform, and draws the following conclusions:

1. The fault line will not form induced voltage before the fault extinction of the fault, and the residual voltage of the line will oscillate and attenuate to 0; After the fault is extinguished, the healthy line will form an induction voltage at the fault line through electrostatic induction, making the voltage of the fault line change from the phenomenon of oscillation attenuation to gradually shift the zero axis;
2. Compared with the automatic reclosing method, this scheme can avoid secondary shocks to the system caused by the DCCB reclosing on permanent faults, and can identify the fault extinguishing moment of the fault, which can improve the speed of power supply restoration to the grid.
3. This scheme uses the line residual voltage and the induced voltage from the non-fault line to the fault line to determine the continuity of the line fault, without additional control.

Given the high probability of single fault occurrences in transmission lines, this study focuses on the arc extinction moment identification method for single fault scenarios. The identification scheme for multiple simultaneous faults on transmission lines requires further in-depth research and will be an important direction for the authors’ subsequent studies.

## Data Availability

All data generated or analysed during this study are included in this published article.
